# Dissecting the Allosteric Fine-Tuning of Enzyme Catalysis

**DOI:** 10.1021/jacsau.3c00806

**Published:** 2024-02-06

**Authors:** Xin-Qiu Yao, Donald Hamelberg

**Affiliations:** † Department of Chemistry, Georgia State University, Atlanta, Georgia 30302-3965, United States; ‡ Department of Chemistry, University of Nebraska Omaha, Omaha, Nebraska 68182-0266, United States

**Keywords:** allosteric regulation, molecular dynamics, kinetics, enzyme catalysis

## Abstract

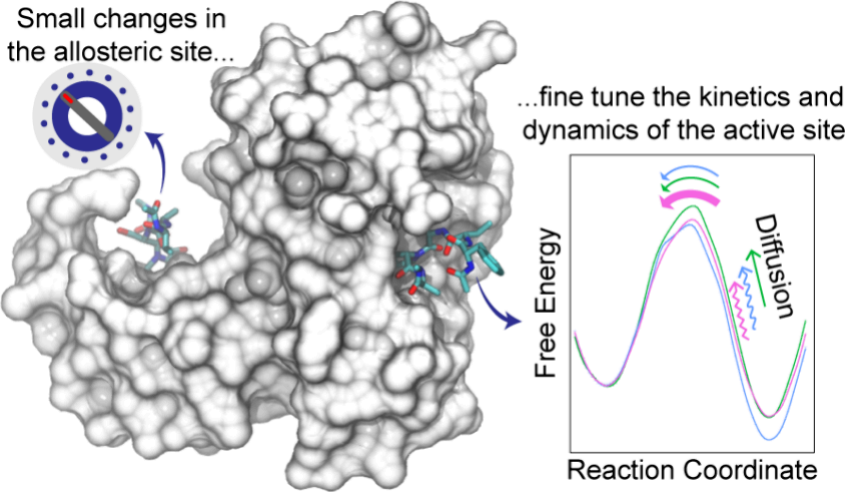

Fully understanding the mechanism of allosteric regulation in biomolecules requires separating and examining all of the involved factors. In enzyme catalysis, allosteric effector binding shifts the structure and dynamics of the active site, leading to modified energetic (e.g., energy barrier) and dynamical (e.g., diffusion coefficient) factors underlying the catalyzed reaction rate. Such modifications can be subtle and dependent on the type of allosteric effector, representing a fine-tuning of protein function. The microscopic description of allosteric regulation at the level of function-dictating factors has prospective applications in fundamental and pharmaceutical sciences, which is, however, largely missing so far. Here, we characterize the allosteric fine-tuning of enzyme catalysis, using human Pin1 as an example, by performing more than half-millisecond all-atom molecular dynamics simulations. Changes of reaction kinetics and the dictating factors, including the free energy surface along the reaction coordinate and the diffusion coefficient of the reaction dynamics, under various enzyme and allosteric effector binding conditions are examined. Our results suggest equal importance of the energetic and dynamical factors, both of which can be modulated allosterically, and the combined effect determines the final allosteric output. We also reveal the potential dynamic basis for allosteric modulation using an advanced statistical technique to detect function-related conformational dynamics. Methods developed in this work can be applied to other allosteric systems.

## Introduction

Allosteric regulation is the primary means for a cell to control diverse processes, including enzyme catalysis, signal transduction, transcription, translation, and metabolism. Underlying allostery is the intramolecular transmission of changes between two sites at a distance, such as the active site of an enzyme and a distal allosteric site. Ligand binding, or other physiochemical modifications such as protonation, posttranslational modification, or mutation, at the distal site modulates the activity of the active site through shifting the conformational ensemble of the entire molecule.^1,2^ Although the physiological consequence of an allosteric modulation is usually dramatic, conformational or dynamic changes associated with the modulation can be subtle and difficult to detect using experimental techniques. As an important complement to experiments, molecular dynamics (MD)^3,4^ has gained increasing interest in understanding allosteric regulation, which provides information about conformational dynamics at a high spatial-temporal resolution. Combining MD simulations with sophisticated statistical techniques and kinetic theory can potentially gain unprecedented insights into the mechanism of allostery.

Many computational studies of allosteric regulation focus on conformational or dynamic changes upon distal perturbations. Direct examinations on changes of reaction kinetics, which are routinely performed in experiments, are much less due to the high computational cost. Also, changes of various factors dictating the kinetics of the catalyzed reaction remain largely unexplored. For example, changes in the active-site conformation and dynamics may affect the energy barrier separating the reactant and the product or alter dynamical properties of the reaction, such as the diffusion coefficient, leading to a modified reaction rate. Separating these effects is a crucial step toward a better understanding of the allosteric fine-tuning of protein function, which is hard to do experimentally and seldom performed computationally.

**Figure 1:**
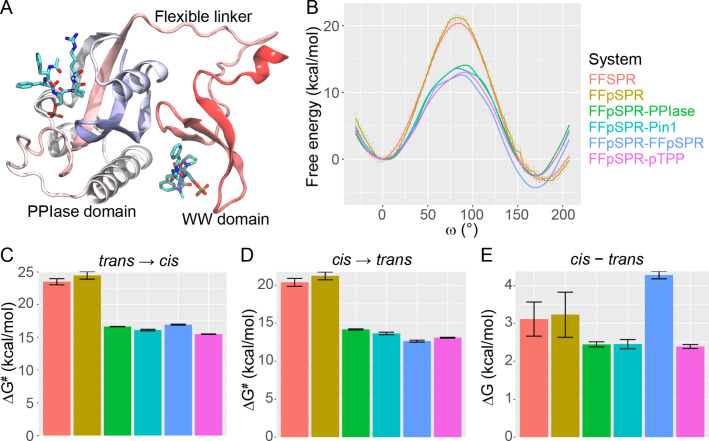
Free energy of prolyl isomerization in free and protein-bound peptides. (A) Structure of Pin1 bound with two peptides in the active site and the WW domain binding site. Pin1 is shown as cartoon color-coded by residue numbers (from red, white, to blue along the chain), whereas peptides are sticks color-coded by atom types. (B) Free energy along the isomerization reaction coordinate (ω). (C, D) Activation energy for the isomerization from *trans* to *cis* and *cis* to *trans*, respectively. Bar heights are defined by the free energy difference between the transition state (∼90°) and *trans* (∼180°) or *cis* (∼0°) in (B). (E) Free energy difference between *cis* and *trans*. See the Materials and Methods for more details of error bars.

This work helps fill the gap by providing details about how allosteric binding fine-tunes factors underlying the reaction rate using the human Pin1 as an example. Pin1 belongs to the Parvulin family of PPIases, a group of enzymes catalyzing the peptidyl-prolyl *cis–trans* isomerization.^5^ The isomerization reaction does not involve bond formation or breakage; hence, it is tractable using classical MD simulations.^6,7^ Unlike other PPIases such as cyclophilins, Pin1 selectively catalyzes substrates with a phosphorylated Ser/Thr (pSer/pThr)-Pro motif.^8,9^ Pin1 is essential for the regulation of cell cycle and a potential therapeutic target for treating diseases including Alzheimer’s and cancer.^10,11^ Pin1 has two domains: the catalytic or PPIase domain and the WW domain that contains two conserved Trp residues (Figure 1A). Both domains bind phosphorylated substrates, but only the PPIase domain catalyzes the prolyl isomerization. The WW domain is assumed to be primarily for substrate binding because of its 10 times stronger binding affinity.^12^ Recent studies^13−17^ have also suggested an allosteric role of the WW domain, stating that substrate binding in this domain affects the binding and catalysis in the distal active site, although details remain poorly understood. Substrates may always bind to both domains of Pin1 in vitro or in vivo, indicating that experiments cannot fully explain the role of the WW domain. In contrast, computational simulations are fully controllable and hence have the power to discern the effect of WW on the PPIase domain under various (substrate binding) conditions.

We have performed extensive MD simulations (totaling 687 μs) to fully characterize the free energy and kinetics of Pin1-catalyzed prolyl isomerization and its allosteric regulation by the WW domain. Here, we adapt a theoretical framework that was previously developed to study enzyme catalysis of human cyclophilin A.^18^ The approach is based on the Kramers’ rate theory,^19^ which allows us to explore various factors underlying the reaction kinetics and their allosteric regulation. Our findings provide new insights into the detailed mechanism of the allosteric fine-tuning of enzyme catalysis, and the tools developed can be valuable for studying other allosteric systems.

## Results and Discussion

We focus on the peptidyl-prolyl isomerization of a known Pin1 peptide substrate, named FFpSPR (Table 1). The reaction kinetics and its underlying free energy surface and diffusion coefficient are calculated and compared for different phosphorylation and enzyme and allosteric effector binding conditions (see Table 2 for the list of systems). Free energy and kinetic data are obtained through restrained (umbrella sampling) and unrestrained MD simulations, respectively, whereas the diffusion coefficient is estimated using an extrapolation scheme based on Kramers’ rate theory. We further examine the potential dynamic basis for the allosteric modulation of the diffusion coefficient using a computational method detecting function-related conformational dynamics. For all analyses, the prolyl amide bond torsion angle (denoted by ω; Figure S1) is chosen as the reaction coordinate. See the Materials and Methods for more details.

**Table 1:** List of Peptides with a Central Prolyl Amide Bond Highlighted in Bold

peptide	sequence	reference
FFSPR	Ac-Phe-Phe-**Ser-Pro**-Arg-Nme^a^	
FFpSPR	Ac-Phe-Phe-**pSer-Pro**-Arg-Nme^b^	(9) and (13)
pTPP	Ac-Ala-Val-Val-Arg-**pThr-Pro**-Pro-Lys-Ser-Pro-Nme	(27)

a All peptides are capped with the acetyl (Ac) and *N*-methylamide (Nme) groups.

b The small “p” indicates that the residue is phosphorylated.

**Table 2:** Definition of Systems

		peptide		
system	protein	PPIase domain or free in solution	WW domain	system size (# of atoms)
FFSPR		FFSPR (free in solution)		7629
FFpSPR		FFpSPR (free in solution)		7680
FFpSPR-PPIase	PPIase domain only (residues 54–163)	FFpSPR		18,692
FFpSPR-Pin1	full-length Pin1	FFpSPR	Apo	38,226
FFpSPR-FFpSPR	full-length Pin1	FFpSPR	FFpSPR	37,720
FFpSPR-pTPP	full-length Pin1	FFpSPR	pTPP	37,457

### Phosphorylation Slows Down Isomerization in Free Peptides

**Figure 2:**
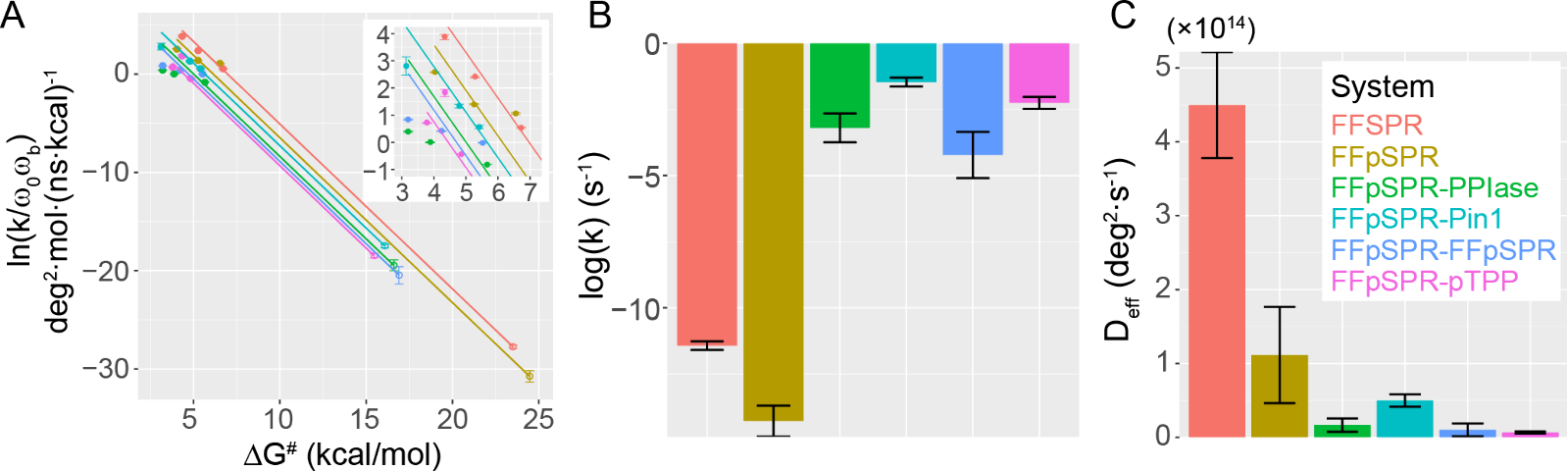
Kinetics and diffusion coefficient of prolyl isomerization in free and protein-bound peptides. (A) Extrapolation of kinetics from modified potentials (filled circles) to the real *V*_2_ (open circles) using the Kramers’ rate theory. Lines are linear fitting using the slope = 1/*k*_B_*T*. Inset: more details about the fitting in the region around the modified potentials. (B, C) Predicted (logarithm of) rate constants and diffusion coefficients, respectively. See the Materials and Methods for more details of error bars.

Phosphorylation raises the energy barrier (Δ*G*^#^) by 1.0 ± 0.8 kcal/mol and 0.9 ± 0.7 kcal/mol for *trans*-to-*cis* and *cis*-to-*trans* transitions, respectively, in peptides free in solution (Figure 1B–D and Table 3). For *trans*-to-*cis*, the phosphopeptide has a catalytic rate (*k*) an order of magnitude slower than the nonphosphorylated peptide (Figure 2A,B and Table 3). Contributions to the slowing of kinetics also include a decrease in the diffusion coefficient (*D*_eff_) upon phosphorylation (Figure 2C and Table 3). However, no significant change is observed in the relative free energy between *cis* and *trans* (Δ*G*), indicating similar *cis* contents for both peptides (Figure 1E and Table 3). Our results are overall consistent with the previous experimental study showing that phosphorylation slowed down isomerization tested on different peptides.^20^ Both the previous and our studies reveal a ∼ 20 kcal/mol energy barrier for the *cis*-to-*trans* isomerization in free peptides. The previous study reported a smaller magnitude of hindrance (0.4 kcal/mol increase in the energy barrier) and a slight increase in the *cis* content by phosphorylation. The small deviations can be explained by the potential peptide sequence dependency of the effect of phosphorylation: in the previous study, the −1 position of peptides is Ala, whereas in our study, the same position has a bulkier side chain (Phe). However, it is also possible that the difference is because of the large uncertainty (0.7 kcal/mol) in our predicted increase in the energy barrier. We do not compare kinetics directly because the previous study only reported the data for the *cis*-to-*trans* transition, whereas this study primarily focuses on the opposite isomerization that usually has a much slower rate due to the lower free energy of the *trans* conformation in free peptides.

**Table 3:** Free Energy, Rate, and Diffusion Coefficient of Prolyl Isomerization in Free and Enzyme-Bound Peptides under Modified and Real Activation Potentials

system	*V*_2_ (kcal/mol)^a^	Δ*G*^#^_trans→cis_ (kcal/mol)	Δ*G*^#^_cis→trans_ (kcal/mol)	Δ*G*_cis-trans_ (kcal/mol)	*k*_trans→ci*s*_ (s^–1^)	*D*_eff_ (deg^2^/s)
FFSPR	7.0 (0.25)	4.33 ± 0.07	2.93 ± 0.11	1.40 ± 0.09	(7.72 ± 0.70) × 10^7^	-c
	8.4 (0.30)	5.28 ± 0.05	3.85 ± 0.10	1.43 ± 0.10	(2.55 ± 0.07) × 10^7^	-
	9.8 (0.35)	6.72 ± 0.05	4.98 ± 0.09	1.74 ± 0.08	(5.02 ± 0.07) × 10^6^	-
	**28.0 (1.00)**^b^	**23.50 ± 0.47**	**20.39 ± 0.51**	**3.11 ± 0.45**	**(1.08 ± 0.17) × 10^–5^**	**(4.50 ± 0.72) × 10^14^**
FFpSPR	7.0 (0.25)	4.02 ± 0.06	2.45 ± 0.10	1.57 ± 0.08	(4.15 ± 0.12) × 10^7^	-
	8.4 (0.30)	5.25 ± 0.07	3.60 ± 0.10	1.65 ± 0.07	(1.75 ± 0.03) × 10^7^	-
	9.8 (0.35)	6.55 ± 0.06	4.66 ± 0.10	1.90 ± 0.09	(1.23 ± 0.04) × 10^7^	-
	**28.0 (1.00)**	**24.47 ± 0.58**	**21.24 ± 0.52**	**3.23 ± 0.60**	**(6.29 ± 3.68) × 10^–7^**	**(1.11 ± 0.65) × 10^14^**
FFpSPR-PPIase	9.8 (0.35)	3.20 ± 0.10	0.62 ± 0.13	2.57 ± 0.11	(7.90 ± 0.24) × 10^6^	-
	11.2 (0.40)	3.88 ± 0.05	1.13 ± 0.07	2.75 ± 0.06	(5.13 ± 0.08) × 10^6^	-
	12.6 (0.45)	5.67 ± 0.13	2.12 ± 0.16	3.55 ± 0.10	(1.91 ± 0.01) × 10^6^	-
	**28.0 (1.00)**	**16.60 ± 0.03**	**14.16 ± 0.07**	**2.45 ± 0.07**	**(4.07 ± 2.21) × 10^–2^**	**(1.66 ± 0.90) × 10^13^**
FFpSPR-Pin1	12.0 (0.43)	3.13 ± 0.12	0.91 ± 0.15	2.22 ± 0.10	(7.45 ± 2.50) × 10^7^	-
	12.6 (0.45)	4.79 ± 0.03	0.58 ± 0.05	4.21 ± 0.04	(1.94 ± 0.14) × 10^7^	-
	14.0 (0.50)	5.41 ± 0.05	2.85 ± 0.07	2.56 ± 0.05	(7.37 ± 0.45) × 10^6^	-
	**28.0 (1.00)**	**16.08 ± 0.12**	**13.63 ± 0.16**	**2.45 ± 0.12**	**(2.30 ± 0.39) × 10^–1^**	**(4.99 ± 0.84) × 10^13^**
FFpSPR–FFpSPR	9.8 (0.35)	3.20 ± 0.08	0.20 ± 0.12	3.00 ± 0.09	(1.09 ± 0.02) × 10^7^	-
	11.2 (0.40)	4.24 ± 0.09	0.17 ± 0.13	4.07 ± 0.09	(6.70 ± 0.09) × 10^6^	-
	12.6 (0.45)	5.52 ± 0.05	0.06 ± 0.08	5.47 ± 0.07	(7.79 ± 0.10) × 10^6^	-
	**28.0 (1.00)**	**16.90 ± 0.08**	**12.60 ± 0.12**	**4.28 ± 0.10**	**(1.46 ± 1.28) × 10^–2^**	**(1.00 ± 0.88) × 10^13^**
FFpSPR-pTPP	12.0 (0.43)	4.34 ± 0.07	1.08 ± 0.10	3.27 ± 0.07	(3.97 ± 0.54) × 10^7^	-
	12.6 (0.45)	3.78 ± 0.07	1.25 ± 0.11	2.53 ± 0.08	(5.17 ± 0.05) × 10^6^	-
	14.0 (0.50)	4.85 ± 0.07	2.36 ± 0.10	2.49 ± 0.08	(3.46 ± 0.03) × 10^6^	-
	**28.0 (1.00)**	**15.46 ± 0.04**	**13.07 ± 0.06**	**2.39 ± 0.05**	**(1.05 ± 0.24) × 10^–1^**	**(6.51 ± 1.47) × 10^12^**

a Values in parentheses are α, i.e., the ratio of the potential over the real potential *V*_2_ = 28 kcal/mol.

b Free energy values and rate constants of free peptides are obtained using the extrapolation scheme. For peptides in complexes, rates are from extrapolation whereas free energy data are calculated by directly using the real potential *V*_2_ = 28 kcal/mol.

c Diffusion coefficient is assumed to be independent of *V*_2_.

### Enzymes Accelerate Isomerization by Orders of Magnitude In Silico

Binding to various Pin1 constructs significantly lowers the energy barrier (by 7.1–9.0 kcal/mol with uncertainty <0.6 kcal/mol) of prolyl isomerization in FFpSPR (Figure 1B–D and Table 3). Accordingly, the enzymes accelerate the rate of isomerization (*trans*-to-*cis*) by 4–6 orders of magnitude (Figure 2A,B and Table 3). The result is consistent with the known typical range of rate enhancements of PPIases. For example, a previous NMR study has revealed that Pin1 accelerates the prolyl isomerization of a phosphorylated peptide derived from the amyloid precursor protein by nearly 10^4^-fold.^21^ Another experimental study using NMR ZZ-exchange has shown an overall *k*_cat_ of Pin1 to be about 1000 s^–1^ using a peptide derived from a human glutamate receptor,^22^ which is 10^6^-fold of the intrinsic rate of prolyl isomerization in free peptides.^20,23^ A similar catalytic power (10^5^-fold) is also found in another PPIase, cyclophilin A.^24,25^

The PPIase domain contributes to most of the enhancement since the domain alone reduces the *trans*-to-*cis* energy barrier by 7.9 ± 0.6 kcal/mol and accelerates the rate by 10^4^-fold (Figure 1B,C, Figure 2A,B, and Table 3). The addition of the WW domain further lowers the barrier by 0.5 ± 0.1 kcal/mol and raises the rate by 6-fold. Previous experimental studies have shown that the PPIase domain alone is functional in vitro,^12,15,26,27^ consistent with the current result. The effect of adding the WW domain is complex and somewhat controversial.^28^ For example, a previous NMR study has clearly shown that the full-length Pin1 is 5- to 8-fold more efficient than a construct containing the PPIase domain only, resembling the current calculation.^26^ However, another study showed a decreasing PPIase activity upon adding WW and supported a negative regulation of function by interdomain contact interactions.^15^ Furthermore, a study by Kern and co-workers suggested that the turnover efficiency was independent of the WW domain.^27^ A limitation of these experiments is the inability to sequester the WW domain from binding substrates. Hence, the previous results do not directly reflect the pure effect of adding the WW domain. As we will discuss later, substrate binding to the WW domain fine-tunes the allosteric effect in a substrate sequence-dependent manner, which may underlie the divergent conclusions of the previous experiments, especially considering that all three previous studies used peptides derived from different target proteins or different sites of the same target. Such allosteric fine-tuning, although subtle, can be biologically significant in the cellular context, as the WW domain is shown to be required in vivo,^12,29^ although such a requirement may simply reflect the essential role of the WW domain as the main interactive module for Pin1 to bind target proteins.

### Ligand Binding in the WW Domain Fine-Tunes Catalysis in the Distal Active Site

The primary energetic modulation by the binding of FFpSPR in the WW domain stabilizes the *trans* conformation of the peptide in the active site. This is evidenced by the 1.1 ± 0.6 kcal/mol increase in the *cis*/*trans* relative free energy compared to the free peptide (Figure 1E and Table 3). Note that both PPIase alone (FFpSPR-PPIase) and full-length Pin1 (FFpSPR-Pin1) have a *cis*/*trans* relative free energy smaller than that in the free peptide, indicating stabilization of the *cis* conformation in the active site (Figure 1E and Table 3). This suggests that ligand binding in the WW domain modulates the conformational preference in the distal active site. Compared to FFpSPR-Pin1, FFpSPR binding in the WW domain raises the energy barrier for the *trans*-to-*cis* transition of the peptide in the active site by 0.8 ± 0.1 kcal/mol, whereas it lowers the barrier for the opposite transition by 1.0 ± 0.2 kcal/mol (Figure 1B–D and Table 3). Accordingly, the *trans*-to-*cis* rate is slowed down by an order of magnitude due to FFpSPR binding in the WW domain (Figure 2A,B and Table 3).

The allosteric effect of peptide binding in the WW domain is peptide sequence-dependent. Binding of the pTPP peptide in WW causes a different effect from that of binding FFpSPR (Figure 1B–E, Figure 2, and Table 3). The *cis*/*trans* relative free energy in the active site is not significantly different between that before and after pTPP binding. The pTPP binding reduces energy barriers for both isomerization directions by 0.6 (uncertainty <0.2) kcal/mol; however, instead of rate enhancement, the binding causes a slight rate reduction for the *trans*-to-*cis* transition by 2-fold. This indicates that free energy is not the only factor under allosteric modulation (see below).

Protein binding reduces the diffusion coefficient by 1–2 orders of magnitude (Figure 2C and Table 3), suggesting a coupling between the protein and the substrate that hinders reaction dynamics. Interestingly, adding the WW domain to the PPIase domain enhances the diffusion coefficient by about 3-fold, further supporting the role of WW in facilitating the catalytic turnover. The increasing diffusion coefficient implies that the interdomain interactions may loosen the coupling between the substrate and the active-site pocket. Again, ligand binding in the WW domain fine-tunes the allosteric effect and reduces the diffusion coefficient by 5- and 8-fold upon binding FFpSPR and pTPP, respectively. The significant reduction of *D*_eff_ by pTPP binding indicates that the negative regulation by the pTPP-WW interaction has a dynamic rather than an energetic source.

A ligand-specific conformational change in Pin1 has been reported in a recent experimental study.^17^ Primarily focusing on two “compact” and “extended” conformational states, the previous study showed peptide-dependent population shifts and allosteric mechanisms. The peptide sequence-dependent allostery in Pin1 has also been explored in our previous study, which mainly examined the substrate binding affinity in the active site.^30^ In the current study, we extend the concept to catalytic activity through exhaustive sampling of the energetics and kinetics of the isomerization process. Especially, our results provide unique insights into the fine-tuning of dynamics in enzyme catalysis by different allosteric effectors. In the following, we further explore the potential dynamic basis of allosteric fine-tuning by identifying function-related conformational dynamics.

### Dynamic Modulation Is Possibly through Shifting the Functional Dynamics of the PPIase Domain

**Figure 3:**
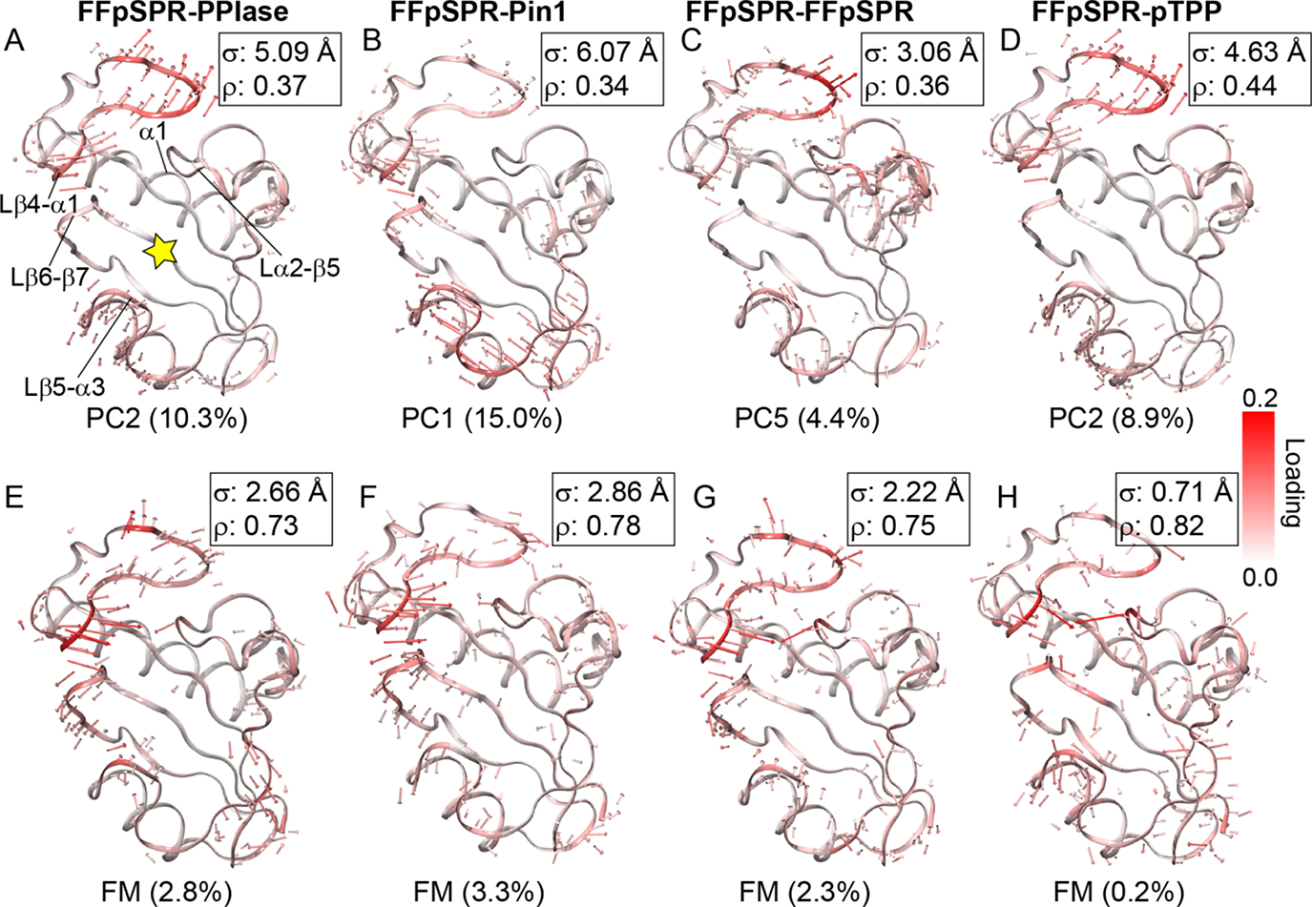
Multiensemble functional mode analysis detects more function-correlated motions than principal component analysis. (A–D) “Porcupine” plots of PCA. The collective motion associated with the PC maximizing the correlation with the reaction coordinate describing the progress of isomerization (ω) is shown as red arrows on the structure of the PPIase domain (shown as cartoon). Lengths and colors of arrows, as well as the color of the structure, are scaled by the loadings of corresponding atoms in the PC axis. Arrows shorter than 1 Å are removed for clarity. Values in parentheses are the percentage of total conformational variance captured by the PC. σ, standard deviation of conformations along the PC. ρ, Pearson’s correlation coefficient between the PC and ω. The substrate’s Pro binding site is indicated by the yellow star in *A*. (E–H) “Porcupine” plots of collective motions detected by multiensemble FMA.

**Figure 4:**
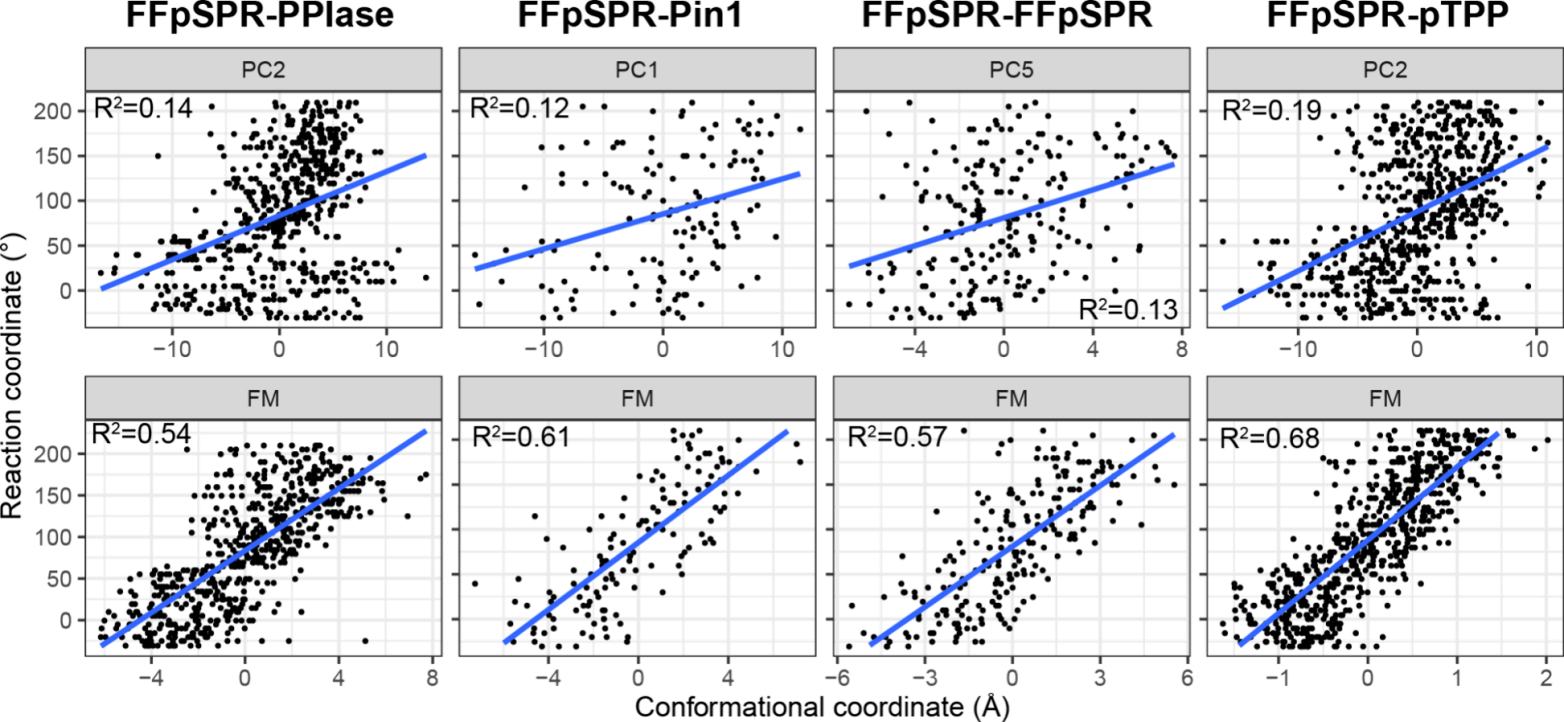
Multiensemble FMA detects motions highly correlated with the progress of reaction. (Top) Correlation between the functional principal component from PCA of each system and the reaction coordinate (reference prolyl ω). Each point represents a conformation from the umbrella sampling. The blue solid line is a linear regression of the data with *R*^2^ shown in the label. (Bottom) Same results using the conformational coordinates mapped onto the functional mode as the abscissa.

To further understand the mechanism of the dynamic modulation in Pin1, we analyze the functional motion of the PPIase domain that is correlated to the progression of the prolyl *cis–trans* isomerization in the active site. An intriguing question here is whether the functional motion is the motion that captures the largest conformational variance. Hence, we first perform (multiensemble) principal component analysis (PCA) using the conformational ensembles generated from different umbrella sampling windows in a single system. The slow slope of the scree plot indicates that significant PCs can be up to the fifth PC (Figure S2). The PC that shows the maximal Pearson’s correlation coefficient (ρ) with the functional variable (the reference ω used for the restraints in umbrella sampling) is picked up for further analysis (denoted as the functional PC), which is not always PC1 (Figure S3). Clearly, PCA shows that collective motions capturing the largest conformational variance are not necessarily functionally relevant, as the correlation between the functional PC and ω is less than 0.5 for all systems (Figure 3A–D and Figure 4). Overall, PCA results are similar among the systems (Figure S4), but functional PCs are distinct, except for FFpSPR-PPIase and FFpSPR-pTPP, both of which choose PC2 as the functional PC (Figure S5). A common feature of the motions described by functional PCs is a twisting motion in the Lβ4−α1 loop surrounding the active site (Figure 3A–D).

The partial least-squares functional mode analysis (FMA)^31^ is adapted to process multiple distinct conformational ensembles (denoted by multiensemble FMA; ensembles used here are the same as in PCA, i.e., those generated by different umbrella sampling windows in a single system). Multiensemble FMA identifies the collective motion that has the highest correlation with ω, showing a correlation greater than 0.7 (Figure 3E–H and Figure 4). The overall pattern of the functional motion is specific to the system and different from those described by PCs (Figure S5 and Figure S6). A consensus functional motion among the systems is a closing motion between Lβ4−α1 and Lα2−β5 loops (Figure 3E–H). Interestingly, FFpSPR-pTPP shows a rather small conformational standard deviation along the functional mode (σ = 0.71 Å) (Figure 3H). The rigidity of FFpSPR-pTPP along the functional mode may underlie the smallest diffusion coefficient of this system (Table 3). Our result supports the important role of protein conformational dynamics in catalyzing the isomerization process^18,32^ and suggests that protein dynamics is a potential medium for the allosteric modulation of the reaction dynamics in the active site.

### Allosteric Regulation by WW Is Likely through Intramolecular Communications

The WW domain can affect catalysis through either intramolecular allosteric communications or binding to the same substrate that binds the active site (the so-called bivalent binding^33^). Without ambiguity, our work supports the hypothesis of intramolecular communications: In our simulation systems, bivalent binding does not exist, but significant allosteric effects are observed. The intramolecular communication hypothesis in Pin1 has also been supported by previous NMR experiments^13−17^ and computational studies.^16,34−36^ Simulation-based computational studies are particularly useful here, as they can help reveal allosteric communication pathways at the atomic level. For example, using simulations, Guo et al. identified two pathways in Pin1 that worked concertedly to propagate the dynamic effect of substrate binding in the WW domain to the active site.^34^ Our previous simulation study also revealed critical interdomain residue–residue contact changes that mediate distinct allosteric effects elicited by the binding of different substrates in the WW domain, which include Trp11-Arg142, Trp11-Glu145, and Pro37-Arg142.^30^ Although details may vary, many experimental and computational studies agree that interdomain interactions are essential for allosteric communication in Pin1.

## Conclusions

We described the detailed mechanism of allosteric fine-tuning of Pin1 catalysis. Allosteric regulation can occur by changing the activation energy or the diffusion coefficient in the prefactor of the catalyzed reaction rate. Our results show that both factors are important and can be modulated allosterically, possibly in an independent manner. Depending on the allosteric effector, these factors can be reduced or enhanced and the combined effect determines the final allosteric output. The modulation of diffusion coefficients is likely through an adjustment of function-related conformational dynamics, supporting the importance of protein dynamics in enzyme catalysis and mediating allosteric regulation. The special example reaction studied here, peptidyl-prolyl isomerization, does not involve covalent bond breakage and formation, which allows us to use classical mechanics for the exploration. Our approach combining simulation-based free energy and kinetics calculations, Kramers’ rate theory, and advanced statistical techniques is general and can be applied to other allosteric systems.

## Materials and Methods

### Software

MD simulations were performed using Amber 16,^37^ molecular modeling by MODELLER 9.12,^38^ and free energy estimation by WHAM 2.0.^39^ Data analysis and visualization were performed using R 3.6,^40^ Bio3D 2.4,^41−43^ and ggplot2 3.4,^44^ unless otherwise indicated. Molecular graphics were rendered by VMD 1.9.^45^ Figures were assembled by using Illustrator 2023 (Adobe, Inc.).

### Preparation of Initial Structures

A total of six systems were constructed including two free peptides and four peptide–protein complexes (Table 2). The peptide, FFpSPR, was used as both a substrate and allosteric effector. This peptide was designed based on the optimal Pin1 substrate identified from a peptide library^9^ and was experimentally well studied.^13^ The other peptide, pTPP, was used as an allosteric effector, which was a truncated version of the peptide derived from a Pin1 substrate (human tau).^27^ For both peptides, the dianionic form of the phosphorylated residue was used as it is more effectively catalyzed by Pin1.^20^

Initial coordinates of free peptides were generated through the LEaP program of Amber 16.^37^ Initial coordinates of Pin1-peptide complexes were from a previous study,^35^ which was based on a crystallographic structure of Pin1 (PDB: 2Q5A(46)). Additional modeling was performed for the N-terminal missing residues (1–6) to generate the full-length Pin1 using MODELLER.^38^ The sequence of the peptide in the complex was edited to match the current study, along with removal of side-chain atoms. Coordinates of peptide side-chain atoms were added back by LEaP. Topology files were prepared using Amber 16 and the ff14SB force field.^47^ PPIase-catalyzed prolyl isomerization is a special case of enzyme catalysis, where classical molecular mechanics is sufficient. First, it is generally accepted that PPIase catalysis does not involve covalent bond breakage or formation.^6^ Second, our previous quantum mechanics calculations showed that the polarization effect during isomerization is small, supporting the use of a fixed-charge force field.^7^ Reoptimized parameters for the torsion angle of the prolyl amide bond were used because they were shown to better describe the energetics of prolyl isomerization than the original AMBER force field.^48^ The original Amber force field files for phosphorylated residues were modified to match atom names and types of ff14SB. Each complex system was solvated in a periodic truncated octahedron box filled with pre-equilibrated TIP3P water,^49^ where each box face was 10 Å away from the surface of solute. Original water molecules from the crystallographic structure were retained. For free peptides, a periodic cubic box was created with each box face 12 Å from the solute. Counter ions (Na^+^ or Cl^–^)^50,51^ were added to neutralize each system.

Prior to production simulations, three rounds of energy minimization were performed for all systems, with each round containing 2000 steps of steepest descent followed by 3000 steps of conjugate gradient. A 500 kcal·mol^–1^·Å^–2^ position restraint was applied to the entire solute and backbone (N, Cα, C, and O) for rounds 1 and 2, respectively,, with the third round having no restraint at all. Each system was then heated up from 100 to 300 K through five rounds (totaling 2.5 ns) of NVT simulations, with each round having a step size of 1 fs, a duration of 500 ps, the Langevin thermostat (collision frequency γ = 1.0 ps^–1^), and a position restraint applied to the solute. The force constants of the restraint were 500, 300, 200, 100, and 50 kcal·mol^–1^·Å^–2^ for rounds 1 to 5. A 1 ns (step size 2 fs) equilibration with no restraint was performed under NPT (300 K, Langevin thermostat; 1 bar, Monte Carlo barostat with coupling constant τ_p_ = 1.0 ps). Long-range electrostatic interactions were treated with particle-mesh Ewald summation^52^ and short-range nonbonded interactions were treated with a 9 Å cutoff. All covalent bonds involving hydrogen atoms were constrained with the SHAKE (nonwater)^53^ or SETTLE (water)^54^ algorithm. Production simulations (for umbrella sampling and kinetics calculations; see below) were performed under the same conditions as equilibration unless otherwise indicated. See Table S1 for the list of all simulations.

### Umbrella Sampling

Umbrella sampling^55,56^ was performed along the reaction coordinate—the dihedral angle (ω) of the prolyl peptide bond defined by atoms Cα and O of the residue preceding the central Pro and Cδ and Cα of the Pro (Figure S1). The dihedral potential of the prolyl bond is expressed by
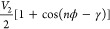
1where *n* is the periodicity, ϕ is the dihedral angle, and γ is the phase angle. In addition to the original *V*_2_ = 28 kcal/mol from the reoptimized parameters,^48^ we performed simulations with smaller *V*_2_ values (i.e., modified potentials with reduced energy barriers) for more efficient sampling of isomerization (see Table S1 for more details). The modified potentials are more tractable in simulating isomerization, which is essential in the kinetic analysis (see below). For each system, 49 windows were generated, each associated with a reference ω ranging from −30 to 210° with an increment of 5°. The initial conformation for each window was generated through a series of additional equilibration simulations. Specifically, starting from 180° (*trans*), a set of MD simulations was performed, each for a duration of 1 ns under a harmonic dihedral restraint on ω with the force constant of 0.06 kcal·mol^–1^·deg^–2^ and the reference angle being ±175°, ±170°, ±165°, etc. The last conformation of the current window was used as the starting point for the next window. Production simulations were performed for 110–560 ns for each window (see Table S1) under a 0.01 kcal·mol^–1^·deg^–2^ harmonic dihedral restraint on ω, with the duration determined by the convergence analysis (see below). A total of 325.4 μs simulation data were generated. The ω angle was saved at every 0.02 ps. Protein conformations were saved every 40 ns for further analyses.

The free energy was obtained using the weighted histogram analysis method (WHAM),^57^ implemented in the WHAM 2.0 program.^39^ The reaction coordinate was divided into 80 bins between −31.5 and 208.5°. The convergence tolerance was set to 10^–6^, and the temperature was 300 K. Errors were estimated by Monte Carlo bootstrap implemented in the WHAM program, with the number of trials being 10 and the random seed 1234. The decorrelation time was set to be 200 ps, which was determined based on the estimated autocorrelation decay time of ω being ∼100 ps (Figure S7) along with the empirical rule of using 2-fold decay time to decorrelate data.

For free peptides, only modified potentials were used for direct umbrella sampling due to the high energy barrier of noncatalyzed isomerization. The free energy of the original potential was obtained through a linear extrapolation scheme similar to previously described.^58^ Specifically, let
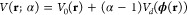
2where α is the scaling factor of the modified potential *V*(**r**;α) [α = 1 is the unmodified potential, *V*_0_(**r**)] and *V*_*d*_ is the unmodified prolyl dihedral potential depending on the angles defining the prolyl torsion, **ϕ**(**r**). We have
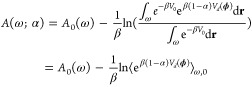
3where *A*(ω; α) and *A*_0_(ω) are the free energies of modified and unmodified potentials, respectively, at the prolyl bond torsion angle ω, β is the inverse of Boltzmann constant times temperature, and ⟨·⟩_ω,0_ is the ensemble average under the unmodified potential and the constraint ω. Using cumulant expansion^59^ up to the first order, we can approximate eq 3 by
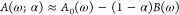
4where *B*(ω) = ⟨*V*_*d*_(**ϕ**)⟩_ω,0_ is an effective potential along the prolyl bond torsion angle. Higher-order terms in eq 4 were ignored because at a fixed ω, the fluctuation of *V*_*d*_(**ϕ**) was small. At each ω, *A*_0_(ω) (and its error) can be obtained as the intercept from a (variance-weighted) linear regression analysis of *A*(ω;α) versus 1 – α. Prior to regression, free energy curves of different α values were superimposed by minimizing the free energy difference at ω = 0 and 180°, where the influence of α was negligible. See Figure S8 for free energy curves of all α values.

The convergence of results was determined by checking the cumulative free energy difference between transition, *cis*, and *trans* states over time (Figures S9–S11). In addition, for each window of the complex systems, we checked the protein–peptide binding stability every 40 ns. If the peptide substrate came out of the active-site pocket, defined by the distance between the Cγ atoms of the central proline of the peptide and the active-site residue His157 being larger than 10 Å, the last 40 ns data were regenerated using the same initial conformation but different initial velocities until the central proline of the peptide always stayed in the active site with the proper orientation.

### Kinetics Analysis

Unrestrained MD simulations of peptidyl-prolyl *cis–trans* isomerization were performed for all systems with modified potentials (reduced energy barriers) (Table S1). All simulations started with the central prolyl amide bond of the peptide (free or in the active site) being in the *trans* conformation. For each system, 100 simulation replicas were performed, which were seeded from a MD simulation with the original dihedral potential at a 1 ns interval. Simulations were kept being extended to generate >90 isomerization events for each system (each replica accounted for at most one event and was not extended once the event was detected). A total of 361.5 μs simulation data were generated, with angular data saved every 0.02 ps.

Isomerization transition was detected by checking whether the ω angle is smaller than the transition-state angle defined by the peak of the free energy curve between the *cis* and *trans* wells (Table S2). Survival probability *S*(*t*) was calculated at every 10 ns from 0 to the latest “escape” time using the Kaplan–Meier method,^60^ with the 95% confidence interval estimated using the exponential Greenwood method (Figure S12).^61^ Single- or multiple-exponential fitting was then performed over *S*(*t*) to obtain the rate constants (and errors). Single-exponential fitting was completed through linear regression in the semilogarithm space using R.^40^ Multiple-exponential fitting was performed by the pracma R package, using the formula
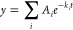
5where *A*_*i*_ and *k*_*i*_ are the coefficient and the rate for the *i*th component, respectively. Data are weighted by the squared confidence interval during both single- and multiple-exponential fitting. The rate constant was directly obtained from the linear regression (single-exponential) or calculated as the weighted average (multiple-exponential)
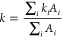
6

Results of the free energy (from umbrella sampling) and kinetics analysis using modified potentials were used to obtain the rate constant under the real activation barrier (*V*_2_ = 28 kcal/mol) through extrapolation. Assuming the framework of Kramers’ theory in the overdamped limit^19^
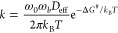
7where *k* is the rate constant, ω_0_ is the curvature of the *trans* well on the free energy curve, ω_b_ is the curvature of the free energy barrier Δ*G*^#^, *k*_B_ is the Boltzmann constant, and *T* is the temperature. *D*_eff_ is the effective diffusion coefficient on the one-dimensional free energy curve and assumed to be independent of *V*_2_. The curvature was calculated by fitting local free energies surrounding a well or barrier to a quadratic function (Table S2). Equation 7 can be expressed in the logarithmic form
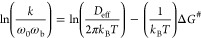
8where (variance-weighted) linear regression was performed for ln(*k*/ω_0_ω_b_) versus Δ*G*^#^ with a well-defined slope of 1/*k*_B_*T*. The intercept of the fitted line produced *D*_eff_, and the line was extrapolated to obtain *k* at Δ*G*^#^ of the real *V*_2_ (which was known from the umbrella sampling).

Error propagation throughout the analysis was performed by using the following formula^62^
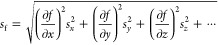
9where *s*_f_ is the standard deviation of a function *f*(*x*, *y*, *z*, ...), *s*_*x*_ is the standard deviation of the variable *x*, *s*_*y*_ is the standard deviation of *y*, and so on.

### Principal Component Analysis

PCA was performed over conformations generated from umbrella sampling. Briefly, the variance-covariance matrix defined by Cartesian coordinates of backbone atoms (N, Cα, C, and O) plus Cβ of the PPIase domain was diagonalized to obtain eigenvalues and eigenvectors. Prior to PCA, conformations were superimposed based on the same group of atoms used for the covariance calculation. Principal component (PC) axes (eigenvectors) were sorted in descending order based on eigenvalues (which also reflect portions of the total conformational variance captured by PCs). Functional PC was defined by the PC having a maximal correlation with the functional variable (ω). The collective motion associated with the functional PC was visualized as a “porcupine” plot mapped on the protein structure, where atomic displacements in the motion were proportional to the loading in the PC. Scree plots, which show the cumulative percentage of variance captured by PC1, PC2, etc., are available in Figure S2.

### Multiensemble Functional Mode Analysis

The original partial least-squares functional mode analysis (PLS-FMA) developed by de Groot and co-workers^31^ was modified to handle multiple ensembles. The reference ω in umbrella sampling was selected as the functional (response) variable. The analysis was to find the collective motion that had the largest correlation with ω. The task is like doing multiple linear regression, except that the explanatory variables were derived from a partial least-squares regression of the degrees of freedom and ω. The same as in PCA, conformations saved from umbrella sampling were superimposed based on backbone and Cβ atoms of the PPIase domain and the same group of atoms were used for the analysis. The number of PLS components (determining the explanatory variables), *n*, was optimized through a 10-fold cross validation, where 9-fold data were used as the training set (to get the FM vector) and the left data as the test set (to calculate the correlation and the error between predicted and reference ω values). The optimal *n* was chosen as the one maximizing the correlation using the test set (Figure S13). Using the optimal *n* and the entire data set, PLS-FMA was reperformed to obtain the final FM. The collective motion represented by the FM was visualized as a “porcupine” plot, in the same way as in PCA.

## Supporting Information Available

List of simulations (Table S1); parameters of free energy curves (Table S2); definition of the reaction coordinate (Figure S1); scree plot of PCA (Figure S2); correlation between PCs and the functional variable (Figure S3); pairwise comparison of PCs from one system to those from another system (Figure S4); comparison of PCA and FMA (Figure S5); overlap between the functional mode from multiensemble FMA and PCs from PCA (Figure S6); example autocorrelation functions of ω in umbrella sampling (Figure S7); free energy curves of (un)modified potentials (Figure S8); convergence of umbrella sampling results for the free peptide FFSPR (Figure S9); convergence of umbrella sampling results for the free peptide FFpSPR (Figure S10); convergence of umbrella sampling results for protein–peptide complexes (Figure S11); survival analysis results (Figure S12); optimization for the multiensemble functional mode analysis (Figure S13) (PDF)

## Notes

Input and parameter files of MD simulations, trajectories, and analysis scripts are available upon reasonable requests to corresponding authors.
