# Supplementary material for: Dissecting the Allosteric Fine-Tuning of Enzyme Catalysis
Source: JACS Au. 2024 Feb 6;4(2):837–46. doi: 10.1021/jacsau.3c00806 (PMC10900222; doi:10.1021/jacsau.3c00806)
Supplement: Supplementary file 1 — au3c00806_si_001.pdf [file au3c00806_si_001.pdf]

Supporting Information

Dissecting the allosteric fine-tuning of enzyme catalysis

Xin-Qiu Yao[1,2]* *and Donald Hamelberg*[1]*

[1]Department of Chemistry, Georgia State University, Atlanta, Georgia 30302-3965, USA.

[2]Department of Chemistry, University of Nebraska Omaha, Omaha, Nebraska 68182-0266, USA.

*Correspondence to: Dr. Xin-Qiu Yao and Prof. Donald Hamelberg; Department of Chemistry, Georgia State University, P. O. Box 3965, Atlanta, GA 30302-3965, USA.

Telephone: (404) 413-5564. E-Mail: xyao@unomaha.edu; dhamelberg@gsu.edu.

Table S1. List of simulations.

System	V_2 (kcal/mol)[a]	Umbrella sampling		Kinetics analysis (*trans*→*cis*)		
		Duration per window (ns)	Total duration (μs)[b]	# of transitions	Avg. duration (ns)	Total duration (μs)[c]
FFSPR	5.6 (0.20)	220	10.78	-[d]	-	-
	7.0 (0.25)	220	10.78	96	100.0	10.00
	8.4 (0.30)	220	10.78	96	107.0	10.70
	9.8 (0.35)	220	10.78	94	223.0	22.30
	11.2 (0.40)	220	10.78	-	-	-
FFpSPR	5.6 (0.20)	220	10.78	-	-	-
	7.0 (0.25)	220	10.78	98	100.0	10.00
	8.4 (0.30)	220	10.78	95	116.0	11.60
	9.8 (0.35)	220	10.78	96	180.0	18.00
	11.2 (0.40)	220	10.78	-	-	-
FFpSPR-PPIase	9.8 (0.35)	110	5.39	99	158.0	15.80
	11.2 (0.40)	430	21.07	96	225.0	22.50
	12.6 (0.45)	120	5.88	92	633.2	63.32
	28.0 (1.00)	520	25.48	-	-	-
FFpSPR-Pin1	12.0 (0.43)	120	5.88	97	123.2	12.32
	12.6 (0.45)	560	27.44	100	282.0	28.20
	14.0 (0.50)	360	17.64	93	301.2	30.12
	28.0 (1.00)	160	7.84	-	-	-
FFpSPR-FFpSPR	9.8 (0.35)	210	10.29	97	120.8	12.08
	11.2 (0.40)	210	10.29	96	172.8	17.28
	12.6 (0.45)	160	7.84	97	157.6	15.76
	28.0 (1.00)	200	9.80	-	-	-
FFpSPR-pTPP	12.0 (0.43)	240	11.76	97	85.6	8.56
	12.6 (0.45)	240	11.76	95	223.2	22.32
	14.0 (0.50)	240	11.76	96	306.4	30.64
	28.0 (1.00)	560	27.44	-	-	-
**Total**			**325.36**			**361.5**

[a]Values in parentheses are α, i.e., the ratio of the potential over the real potential V_2=28 kcal/mol.

[b]Each umbrella sampling calculation has 49 windows.

[c]A total of 100 simulation replicas were performed for each system.

[d]Data not available because the system was not used for kinetic analysis.

Table S2. Parameters of free energy curves.

System	V_2 (kcal/mol)[a]	Transition state angle (°)	ω_0 ($kcal^{1/2} \cdot mol^{-1/2} \cdot deg$)	ω_b ($kcal^{1/2} \cdot mol^{-1/2} \cdot deg$)
FFSPR	7.0 (0.25)	78	0.046	0.035
	8.4 (0.30)	87	0.047	0.049
	9.8 (0.35)	81	0.049	0.060
	28.0 (1.00)[b]	-[c]	0.099	0.121
FFpSPR	7.0 (0.25)	75	0.049	0.065
	8.4 (0.30)	78	0.060	0.072
	9.8 (0.35)	78	0.055	0.078
	28.0 (1.00)	-	0.106	0.134
FFpSPR-PPIase	9.8 (0.35)	93	0.086	0.062
	11.2 (0.40)	90	0.076	0.068
	12.6 (0.45)	87	0.087	0.050
	28.0 (1.00)	-	0.118	0.097
FFpSPR-Pin1	12.0 (0.43)	99	0.064	0.070
	12.6 (0.45)	96	0.084	0.061
	14.0 (0.50)	93	0.053	0.079
	28.0 (1.00)	-	0.108	0.083
FFpSPR-FFpSPR	9.8 (0.35)	87	0.067	0.071
	11.2 (0.40)	84	0.087	0.051
	12.6 (0.45)	96	0.091	0.087
	28.0 (1.00)	-	0.111	0.101
FFpSPR-pTPP	12.0 (0.43)	90	0.071	0.091
	12.6 (0.45)	87	0.069	0.037
	14.0 (0.50)	90	0.075	0.071
	28.0 (1.00)	-	0.107	0.103

[a]Values in parentheses are α, i.e., the ratio of the potential over the real potential V_2=28 kcal/mol.

[b]Free energy curves of free peptides with V_2=28 kcal/mol are obtained using the extrapolation scheme.

[c]Systems with V_2=28 kcal/mol were not used for survival analysis.

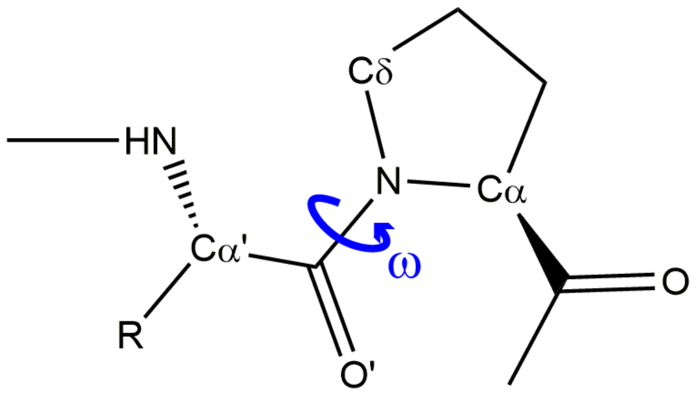

Figure S1. Definition of the reaction coordinate. The peptidyl prolyl ω torsion angle (defined by Cα’-O’-Cδ-Cα) is used as the reaction coordinate.

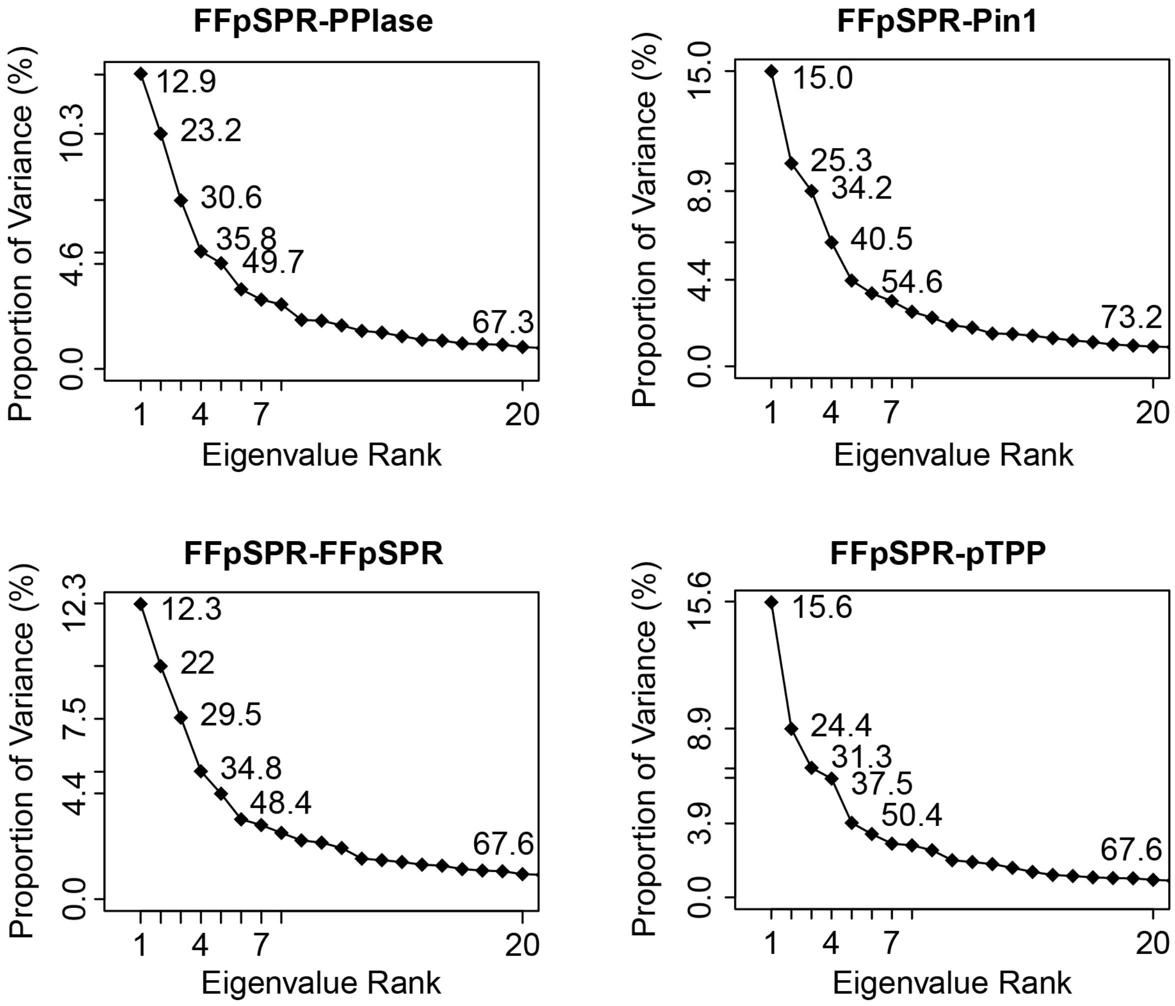

Figure S2. Scree plot of PCA.

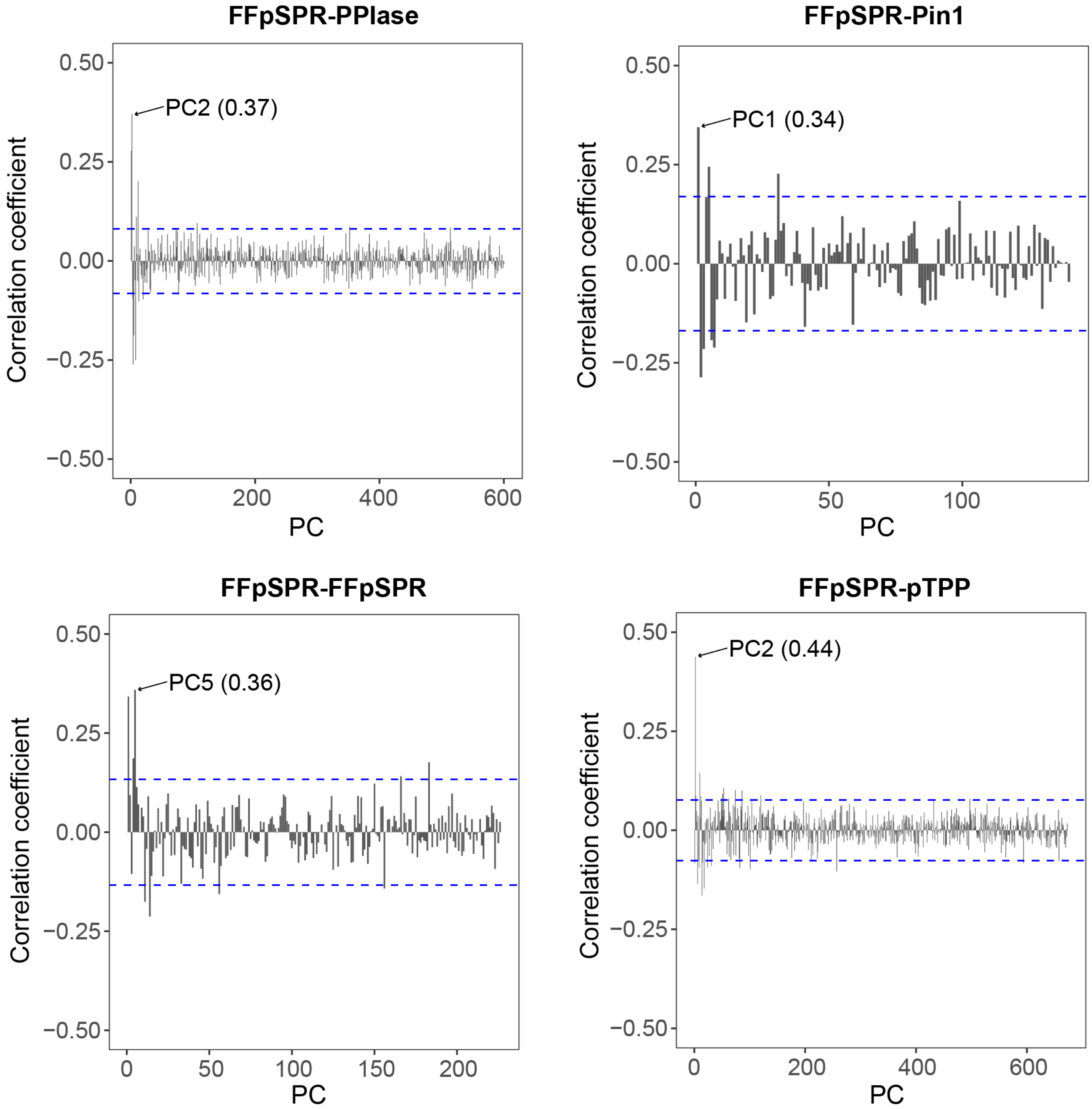

Figure S3. Correlation between PCs and the functional variable. Pearson's correlation coefficient is shown between each PC and the reference prolyl peptide bond torsion angle, ω. The number of PCs is determined by the number of conformations used for PCA, which varies among systems. The maximal correlation and its associated PC are indicated. Blue dashed lines are twice the standard deviation of correlations.

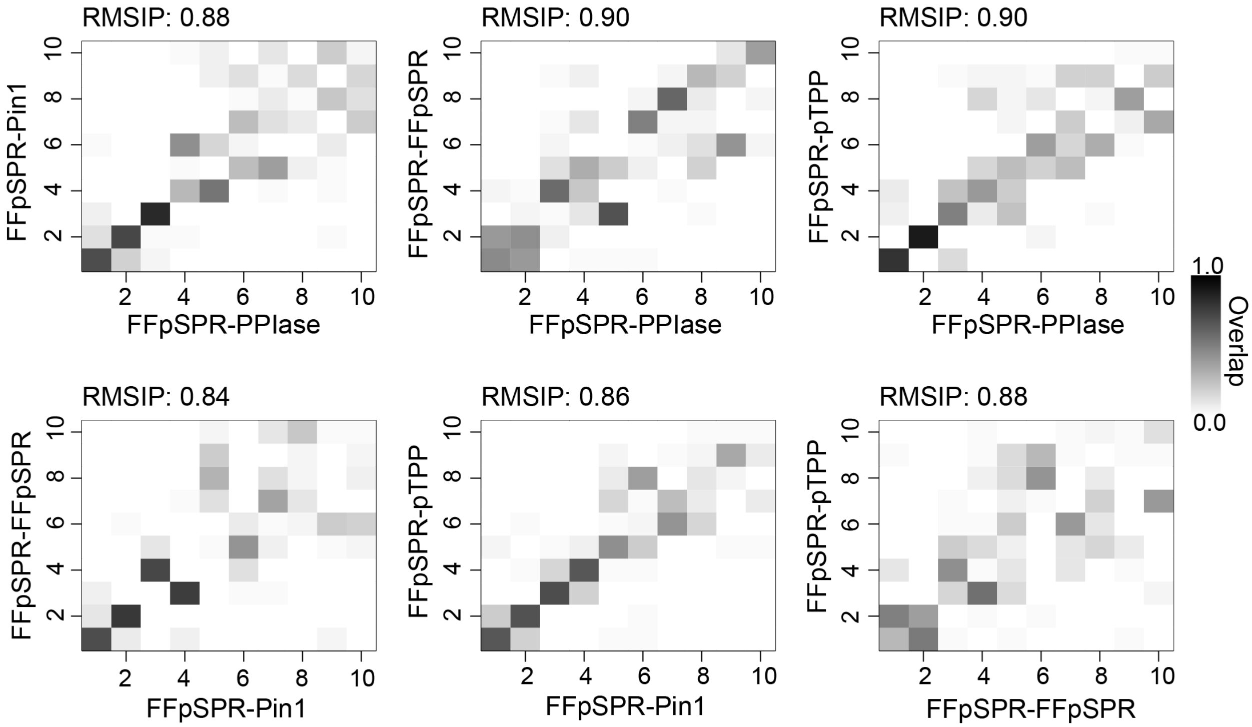

Figure S4. Pairwise comparison of PCs from one system to those from another system. The overlap of each pair of PCs is defined by the squared dot product of the PC axes and is color coded by the gray scale. Root mean square inner product (RMSIP)[1] for the top 10 PCs is calculated to compare the overall similarity of the two sets of PCs.

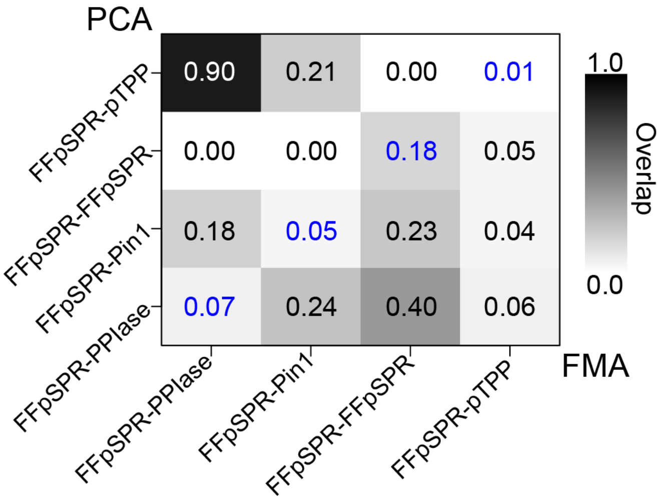

Figure S5. Comparison of PCA and FMA. The overlap (squared dot product) between functional PC axes from PCA of different systems is shown in the upper triangular. Functional PC is defined as that maximizes the correlation with the functional variable (ω), which are PC2, PC1, PC5, and PC2 for FFpSPR-PPIase, FFpSPR-Pin1, FFpSPR-FFpSPR, and FFpSPR-pTPP, respectively. A similar result for functional modes from multi-ensemble FMA is shown in the lower triangular. The diagonal shows the comparison between the PC and the functional mode from the same system.

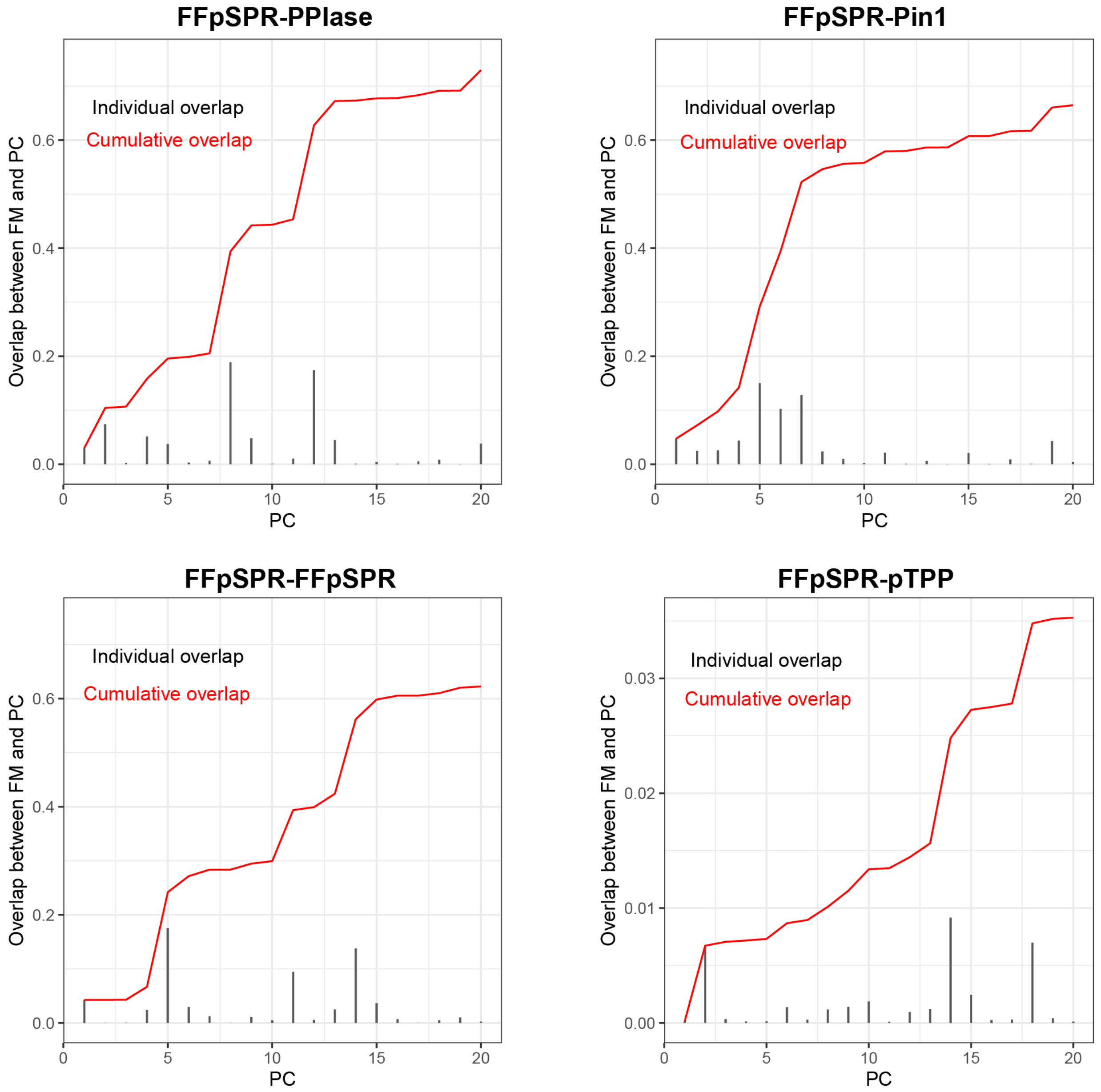

Figure S6. Overlap between the functional mode from multi-ensemble FMA and PCs from PCA. Overlap is defined by the squared dot product between FM and PC vectors. Both individual (black bars) and cumulative (i.e., sum of preceding overlap values; red lines) overlaps are shown.

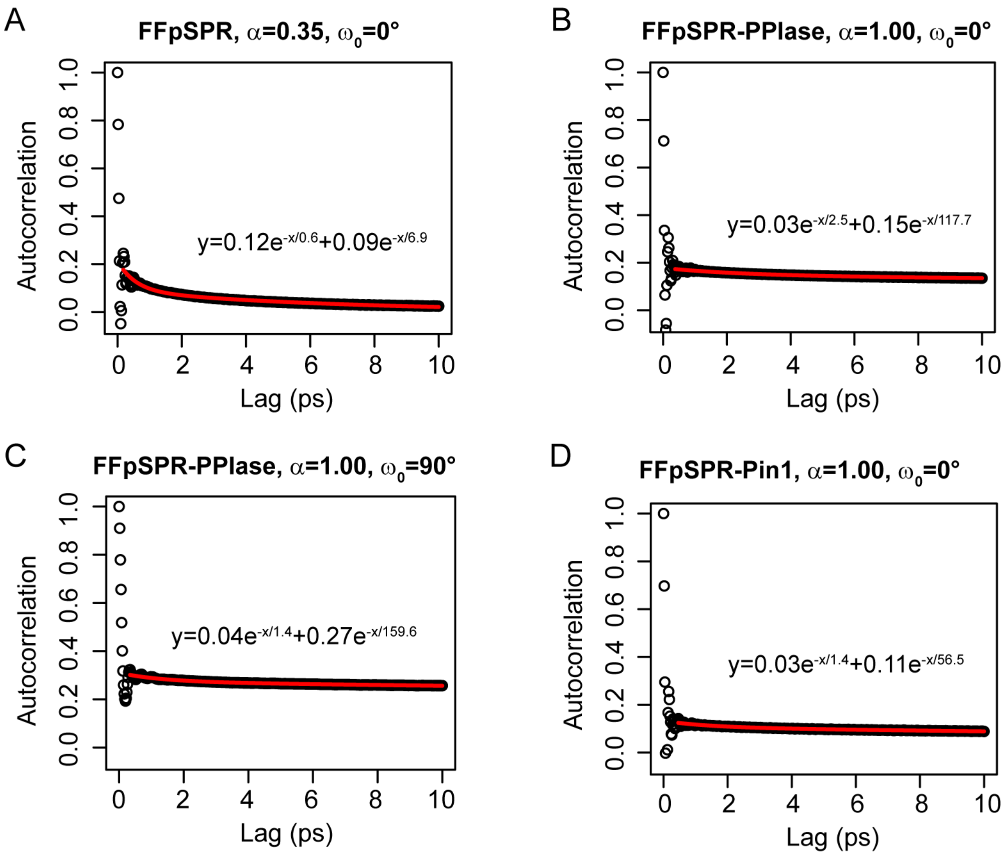

Figure S7. Example autocorrelation functions of ω in umbrella sampling. The sampling of ω in four systems is examined. (*A*) Free phosphorylated peptide with modified potential (α=0.35) and reference angle ω_0=0° (*cis*). (*B*) FFpSPR bound in isolated PPIase domain with unmodified potential (α=1.00) and reference angle ω_0=0° (*cis*). (*C*) FFpSPR bound in isolated PPIase domain with unmodified potential (α=1.00) and reference angle ω_0=90° (TS). (*D*) FFpSPR bound in full-length Pin1 with unmodified potential (α=1.00) and reference angle ω_0=0° (*cis*). For all data, the correlation drops to a low value (<0.3) very quickly (within a few ps). After the fast phase, there is a short "oscillation" phase before reaching a slow phase. Double-exponential fitting is performed for the slow phase (red lines and texts showing the fitted equations). The free peptide has the fastest decay time (6.9 ps in the slower mode). Within the PPIase domain, the decay of autocorrelation slows down, with decay time being 56.6-159.6 ps in the slower mode.

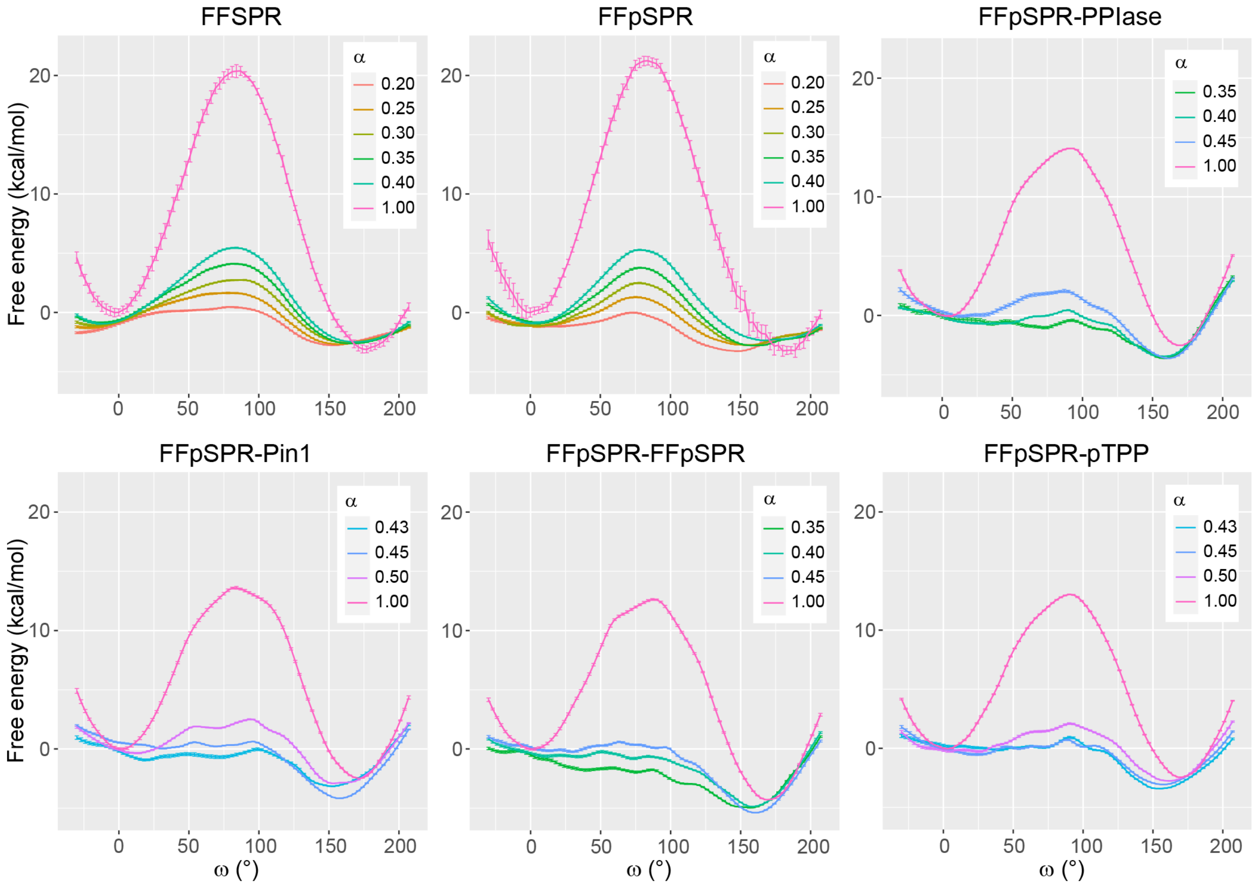

Figure S8. Free energy curves of (un)modified potentials. Free energies are color coded by the parameter of the (un)modified potential, α. All data are obtained from direct umbrella sampling calculations except for unmodified (α=1.00) free peptides (FFSPR and FFpSPR), whose free energies are obtained from extrapolations of free energies of modified potentials. Error bars represent the standard error of the mean.

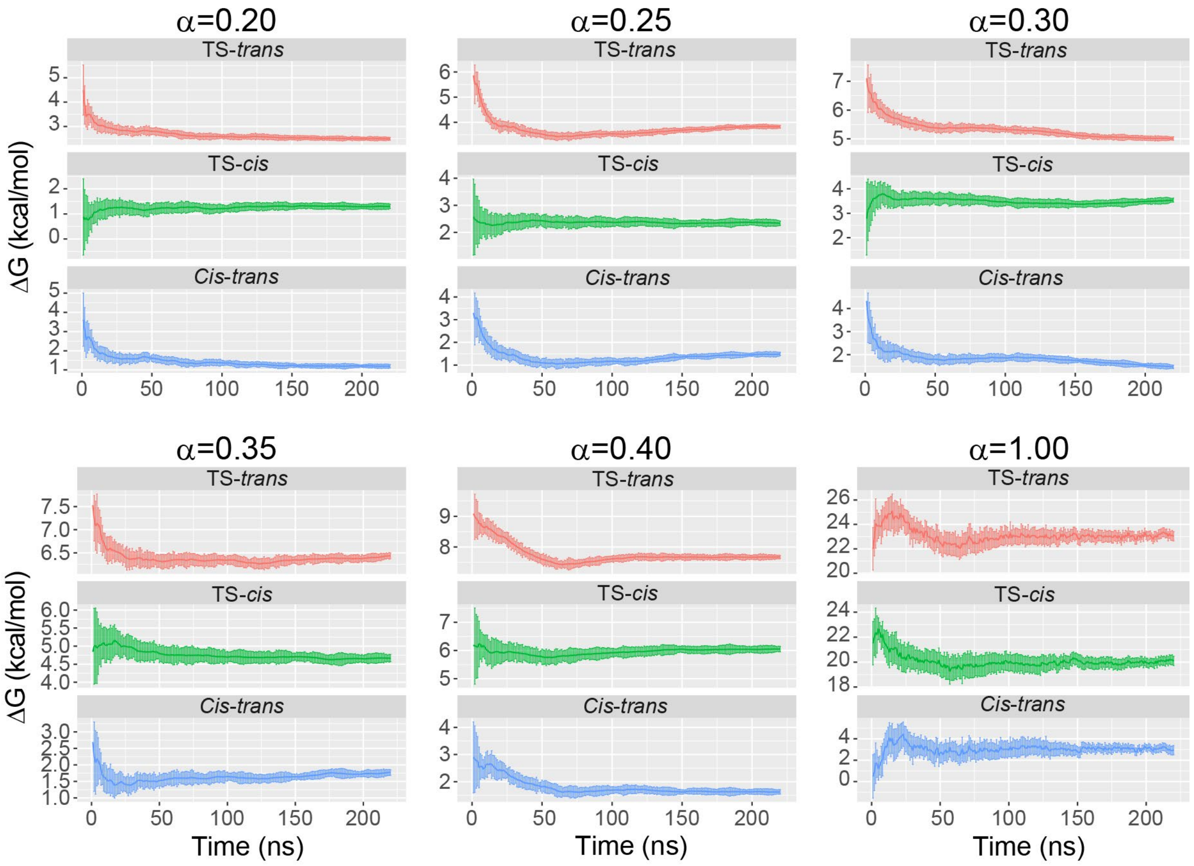

Figure S9. Convergence of umbrella sampling results for the free peptide FFSPR. Cumulative free energy differences between the transition state (TS), *cis*, and *trans* under different α values are shown.

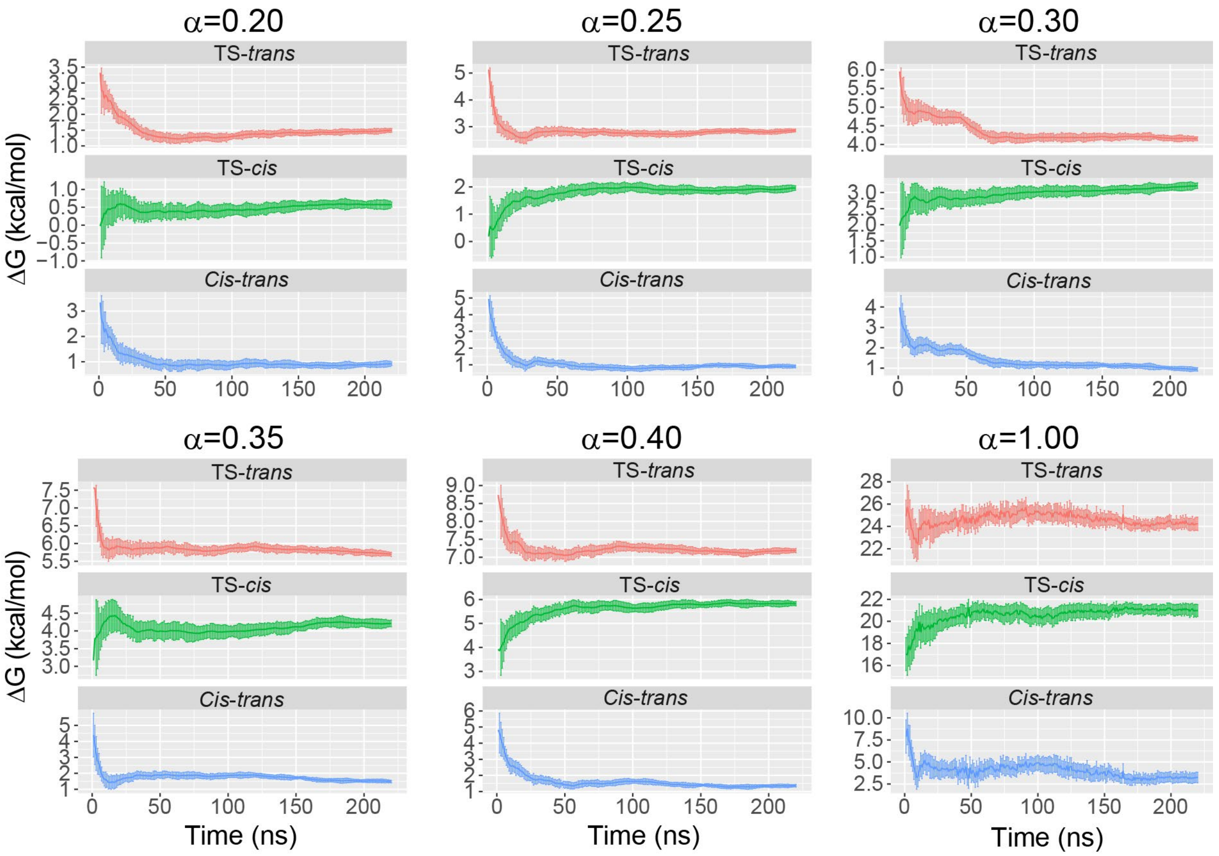

Figure S10. Convergence of umbrella sampling results for the free peptide FFpSPR. Cumulative free energy differences between the transition state (TS), *cis*, and *trans* under different α values are shown.

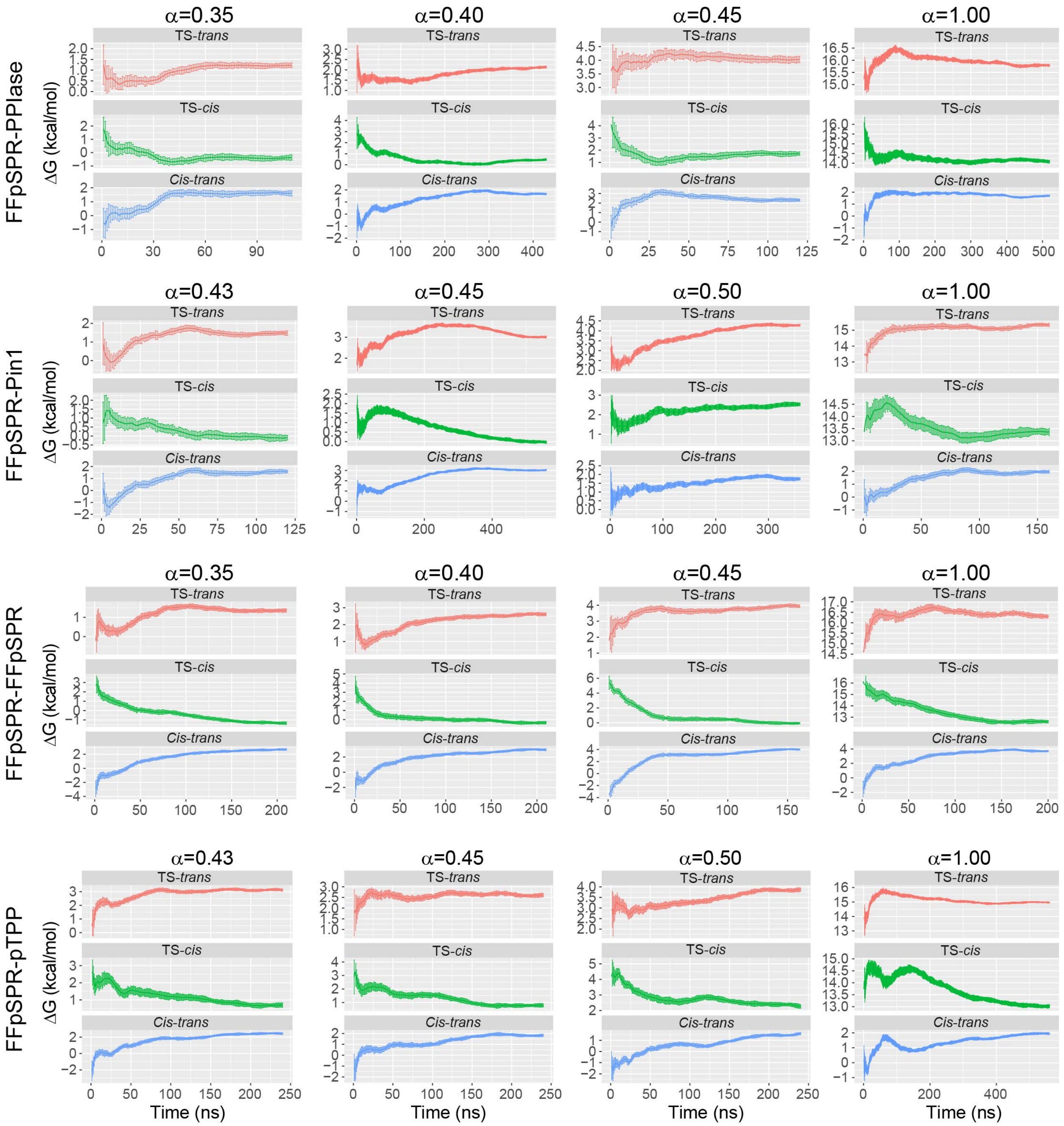

Figure S11. Convergence of umbrella sampling results for protein-peptide complexes. Cumulative free energy differences between the transition state (TS), *cis*, and *trans* under different α values are shown.

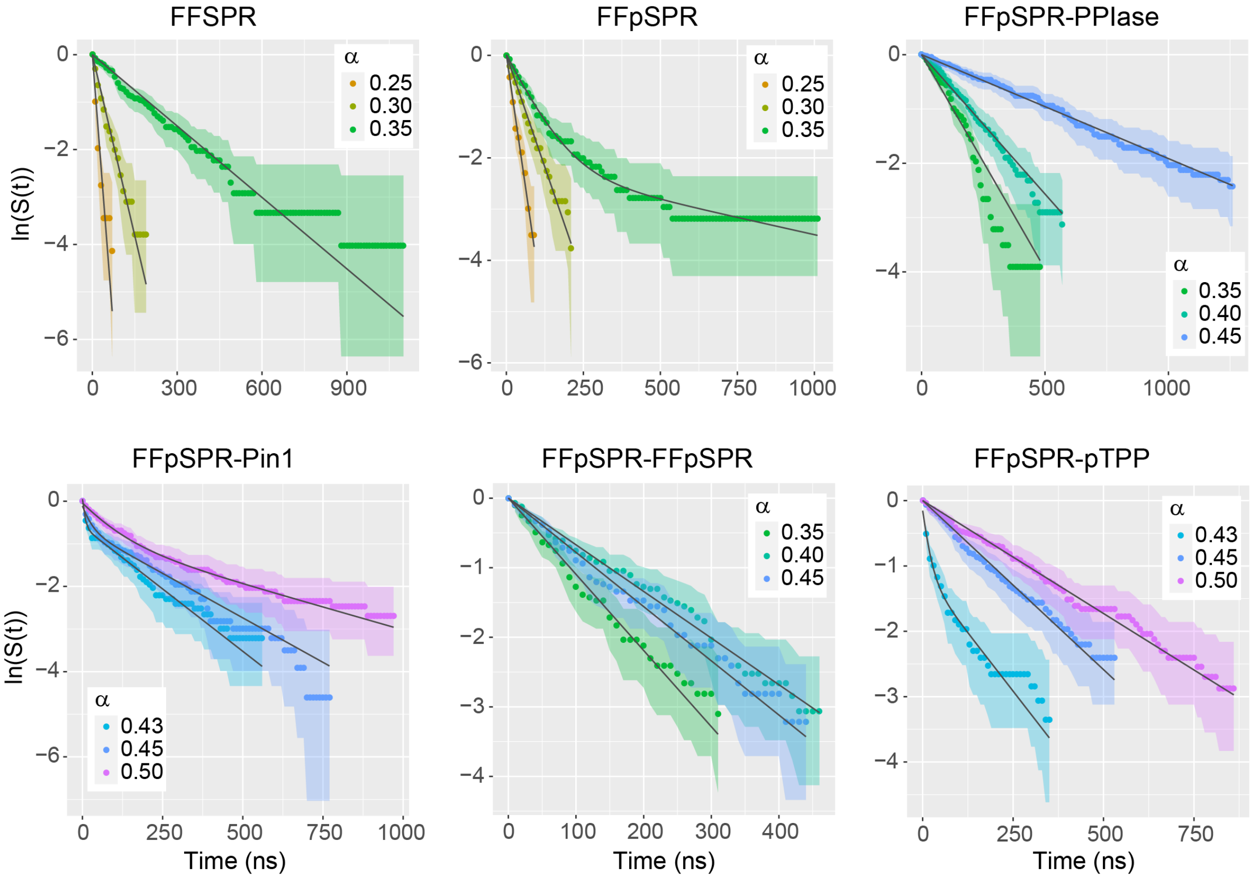

Figure S12. Survival analysis results. Survival probability *S(t)* is shown as points color coded by the parameter of the modified potential, α. The 95% confidence interval is shown as shaded areas color coded the same as points. Single- or multiple-exponential fittings are shown as solid lines.

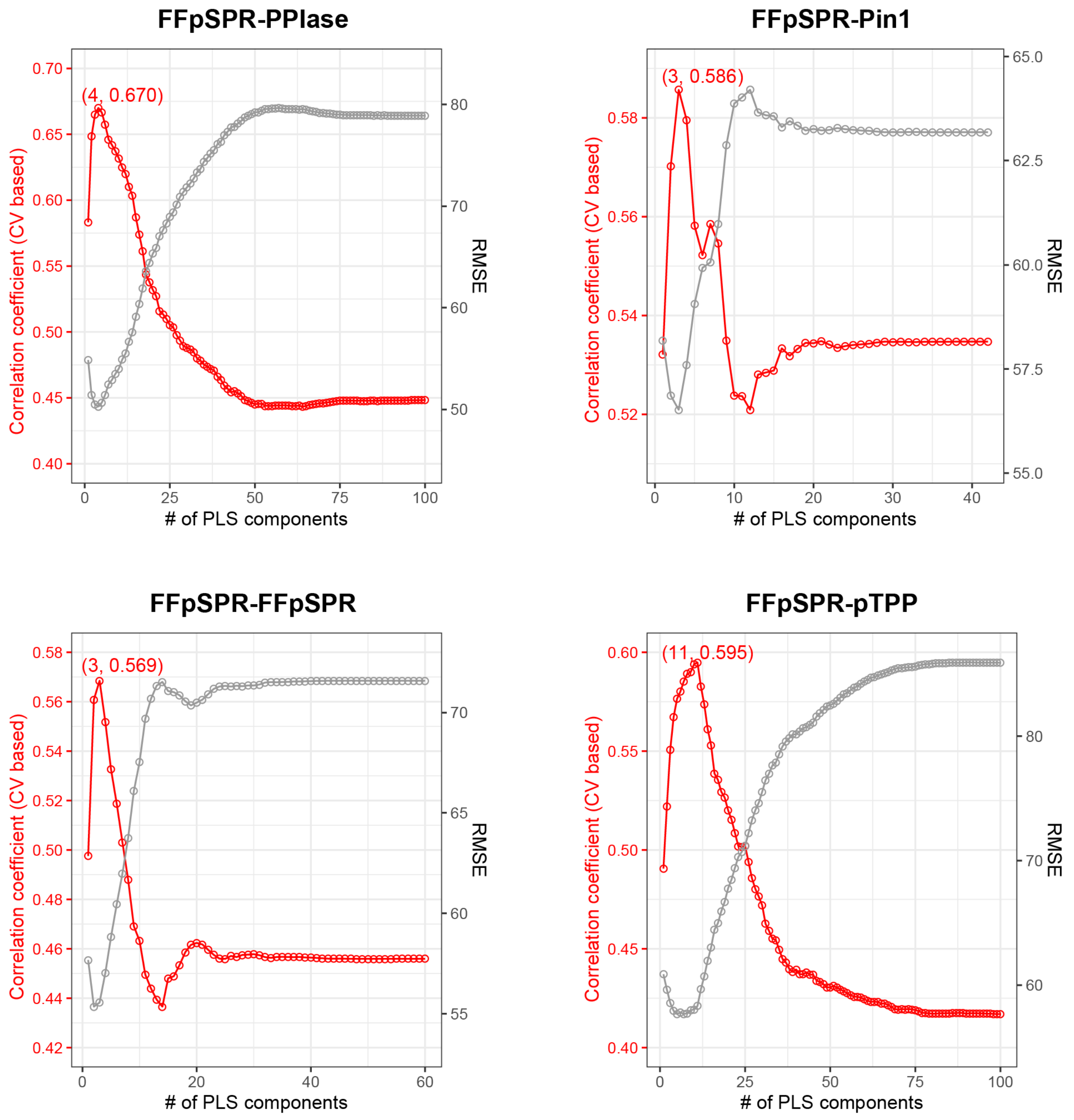

Figure S13. Optimization for the multi-ensemble functional mode analysis. The number of partial least-squares (PLS) components (*n*) is optimized to be the one that maximizes the correlation between the functional mode and the functional variable (i.e., the progression of the prolyl isomerization, ω; red line and circles) through cross validation (CV) (See Methods for more detail). For convenience, the root mean square error (RMSE; gray line and circles), which describes the difference between predicted and observed ω values, is also shown (unit: °). Values in parentheses in the labels are the optimal *n* and the associated correlation, respectively.

Supplemental References

1. Amadei, A.; Ceruso, M. A.; Di Nola, A., On the convergence of the conformational coordinates basis set obtained by the essential dynamics analysis of proteins' molecular dynamics simulations. *Proteins* **1999,** *36*, 419-424.
